# Ontogenetic shift or not? Different foraging trade‐offs within the meso‐ to bathypelagic fish community

**DOI:** 10.1002/ece3.11129

**Published:** 2024-03-20

**Authors:** Liz Loutrage, Anik Brind'Amour, Tiphaine Chouvelon, Jérôme Spitz

**Affiliations:** ^1^ Observatoire Pelagis UAR 3462 La Rochelle Université/CNRS La Rochelle France; ^2^ DECOD (Ecosystem Dynamics and Sustainability: From Source to Sea), Ifremer, Institut Agro, INRAE Nantes France; ^3^ Centre d'Etudes Biologiques de Chizé (CEBC) UMR 7372 La Rochelle Université/CNRS Villiers‐en‐Bois France; ^4^ Ifremer CCEM Contamination Chimique des Écosystèmes Marins Nantes France

**Keywords:** body size, deep pelagic, stable isotopes, trophic ecology, variance partitioning

## Abstract

During ontogeny, the increase in body size forces species to make trade‐offs between their food requirements, the conditions necessary for growth and reproduction as well as the avoidance of predators. Ontogenetic changes are leading species to seek out habitats and food resources that meet their needs. To this end, ontogenetic changes in nocturnal habitat (vertical use of the water column) and in the type of food resources (based on stable isotopes of nitrogen) were investigated in 12 species of deep pelagic fish from the Bay of Biscay in the Northeast Atlantic. Our results revealed the existence of major differences in the ontogenetic strategies employed by deep pelagic fishes. Some species showed ontogenetic changes in both vertical habitat use and food resources (e.g. Jewel lanternfish (*Lampanyctus crocodilus*) and Atlantic soft pout (*Melanostigma atlanticum*)). In contrast, other species showed no ontogenetic change (e.g. Koefoed's searsid (*Searsia koefoedi*) and Lancet fish (*Notoscopelus kroyeri*)). Some species only changed food resources (e.g. Spotted lanternfish (*Myctophum punctatum*), Spotted barracudina (*Arctozenus risso*) and Stout sawpalate (*Serrivomer beanii*)), while others seemed to be influenced more by depth than by trophic features (e.g. Bluntsnout smooth‐head (*Xenodermichthys copei*) and Olfer's Hatchetfish (*Argyropelecus olfersii*)). These results suggest that to meet their increasing energy requirements during ontogeny, some species have adopted a strategy of shifting their food resources (larger prey or prey with a higher trophic level), while others seemed to maintain their food resources but are most likely increasing the quantity of prey ingested. As fish species can have different functional roles during their development within ecosystems, characterising ontogenetic changes in mesopelagic fish species is a crucial step to be considered in future research aimed at understanding and modelling the complexity of deep‐pelagic food webs.

## Graphical abstract

The community of deep‐water pelagic fishes in the Bay of Biscay has shown a great diversity of ontogenetic change strategies. Some species have undergone changes in both habitat use (use of the water column) and food sources (changes in δ^15^N values), while others have undergone only one of these changes. Finally, some species did not appear to have undergone any ontogenetic changes.

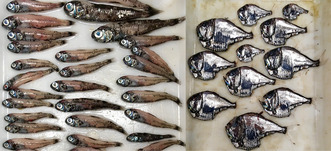

## 1 INTRODUCTION

Ontogenetic shifts in marine predators are major drivers in the mechanisms underlying ecosystem structure and functioning (Nakazawa, 2015; Rudolf & Rasmussen, 2013). They are also considered a determinant of food web diversity and stability, community resilience and responses to disturbance (de Roos & Persson, 2013; Nakazawa, 2015; Nilsson et al., 2018). Although the importance of these ubiquitous changes in ecosystems is well established, community ecology has traditionally been based on species, thereby erasing intraspecific differences. During ontogeny, individuals must make trade‐offs between their dietary needs, conditions necessary for reproduction and predator avoidance (Kimirei et al., 2013; Sánchez‐Hernández et al., 2019; Sutherland, 1996). All these needs and trade‐offs change over the lifetime of species, requiring them to find habitats that meet their needs (Ludwig & Rowe, 1990; McNamara & Houston, 1986; Werner & Gilliam, 1984; Werner & Hall, 1988). Thus, shifts in diet and habitat use during ontogeny can lead to segregation in the niches occupied by individuals of a species and thus reduce intraspecific competition within a population (Sánchez‐Hernández & Cobo, 2012; Wollrab et al., 2013). At the interspecific level, these shifts also play an important role in competitive interactions and niche partitioning (de Roos & Persson, 2013; Woodward et al., 2005; Woodward & Hildrew, 2002).

Fish species often show a close relationship between body size, which is generally related to the size of the mouth opening, and the size of the prey they consume (Dunic & Baum, 2017). Therefore, ontogenetic shifts in resource use are very common in fish (Werner & Gilliam, 1984). In general, early‐stage fish feed on phytoplankton, zooplankton or small invertebrates (Nunn et al., 2012). As their vision and swimming performance improve, fish begin to feed on macroinvertebrates and fish (Huss et al., 2013). These shifts in food types are often associated with or caused by a shift in habitat use (Sánchez‐Hernández et al., 2019; Werner & Gilliam, 1984). For instance, a change in diet may be the consequence of a change in habitat to cope with new predation risks during ontogeny, or it may be caused by the search for more nutritious and/or more abundant prey (Sánchez‐Hernández et al., 2019). An example of the consequences of a change in diet dictated by a change in habitat use is that of small individuals of the Gobiidae species *Pterogobius elapoides*, feeding on abundant pelagic copepods in the water column where predation is high. As the individuals grow larger, they limit the risk of predation by feeding only on prey found in the sediments of the sandy bottom (Choi & Suk, 2012).

Meso‐ and bathypelagic fish communities (i.e. inhabiting the mesopelagic zone between 200 and 1000 m, and the bathypelagic zone below 1000 m depth) are believed to dominate the fish biomass worldwide (Irigoien et al., 2014). The deep pelagic food web is supported solely by phytoplankton primary production, resulting in the segregation of deep pelagic fish trophic niches essentially along a continuum of trophic levels (Chouvelon et al., 2022; Richards et al., 2023; Stowasser et al., 2012; Valls et al., 2014). Three main food guilds are generally described for midwater fishes: zooplanktivores (e.g. Myctophidae), micronektivores (e.g. Stomiidae) and generalists (e.g. Eurypharyngidae, in which a wide variety of prey even benthic, is found) (Drazen & Sutton, 2017; Gartner et al., 1997). In addition to these guilds, two main foraging strategies are employed by deep pelagic fishes. Part of the community performs diurnal vertical migrations (DVM) at night from the mesopelagic to the epipelagic zone to feed (Badcock & Merrett, 1976; Clarke, 1963; Watanabe et al., 1999). This migratory behaviour is very energy‐consuming but is compensated by the high prey density in the epipelagic zone and the reduction of visual predation at night. The non‐migratory part of the community remains at depth. The non‐migratory species thus live in an environment of low prey density but have lower energy requirements and a low risk of predation (Herring, 2001; Marshall, 1979).

Most deep pelagic species, particularly Myctophidae, spend their larval stage in the productive epipelagic zone (Ahlstrom, 1959; Bowlin, 2016; Loeb, 1979; Moser & Smith, 1993; Sassa et al., 2002, 2004). Within species, individual size generally increases with depth, indicating ontogenetic vertical migrations (Badcock & Araujo, 1988; Kawaguchi & Mauchline, 1982; Loeb, 1979; Sassa & Kawaguchi, 2006). This ontogenetic shift along the vertical habitat is related to shifts in morphology and pigmentation (i.e. individuals becoming darker, having photophores and well‐developed musculature) (Moser, 1996). Similarly, the adults of several species have a different depth distribution according to size, with larger individuals at deeper depths (Auster et al., 1992; Badcock & Merrett, 1976; Fanelli et al., 2014; Loeb, 1979; Sassa et al., 2007; Stefanescu & Cartes, 1992; Willis & Pearcy, 1980). These ontogenetic shifts in habitat use may be related to shifts in diet, as in the case of *Lampanyctus crocodilus*, where senescent adults stop migrating and adopt benthopelagic behaviour by feeding on epibenthic prey (Fanelli et al., 2014; Stefanescu & Cartes, 1992).

Intraspecific trophic changes can be monitored from stable isotope signatures (Hammerschlag‐Peyer et al., 2011; Layman et al., 2012). For decades, stable isotope ratios of nitrogen (δ^15^N values) have been widely used as an indicator of species' trophic level (Drazen & Sutton, 2017; Peterson & Fry, 1987; Zanden & Rasmussen, 2001). This is because nitrogen isotopes undergo a significant and relatively predictable level of fractionation during trophic transfer between a predator and its prey, leading to a difference in δ^15^N values (~3‰–5‰) between two theoretical trophic levels and allowing the relative trophic level of species to be inferred from their δ^15^N values (Hussey et al., 2014; Peterson & Fry, 1987; Post, 2002). Since the pelagic ecosystem has a wide depth gradient, microbial degradation of organic matter in suspended particles also influences δ^15^N values, with increasing values with depth (Casciotti et al., 2008; Saino & Hattori, 1980). An enrichment in ^15^N is thus found in zooplankton at greater depths (Hannides et al., 2013; Koppelmann et al., 2009) and in deep benthic communities (Bergmann et al., 2009; Trueman et al., 2014).

Only a few studies examined the effect of species size and depth on δ^15^N values of deep pelagic fish, such as in the Iberian Peninsula (North‐East Atlantic) and the Gulf of Mexico (North‐West, Atlantic) (Richards et al., 2023; Romero‐Romero et al., 2019), but never at the intraspecific scale. Here, we aimed to quantify the intraspecific influence of body size on nocturnal habitat use and trophic ecology for 12 deep pelagic fish belonging to nine genera, including both migratory and non‐migratory species from the Bay of Biscay, NE Atlantic. Differences in nocturnal body size distribution within each species were deduced from trawling data, while trophic shifts were studied using stable isotopes of nitrogen measured in fish muscle tissues. The first objective was to investigate if a shift in body size with depth (i.e. relationships between individual size and sampling depth) is observed at both the intraspecific and community level. The second objective was to explore if a shift in the trophic ecology is also observed in relation to body size (from the measurement of δ^15^N values). To this end, the influence of individual size and/or sampling depth on δ^15^N values was quantified for each species.

## 2 MATERIALS AND METHODS

### 2.1 Sampling

**FIGURE 1:**
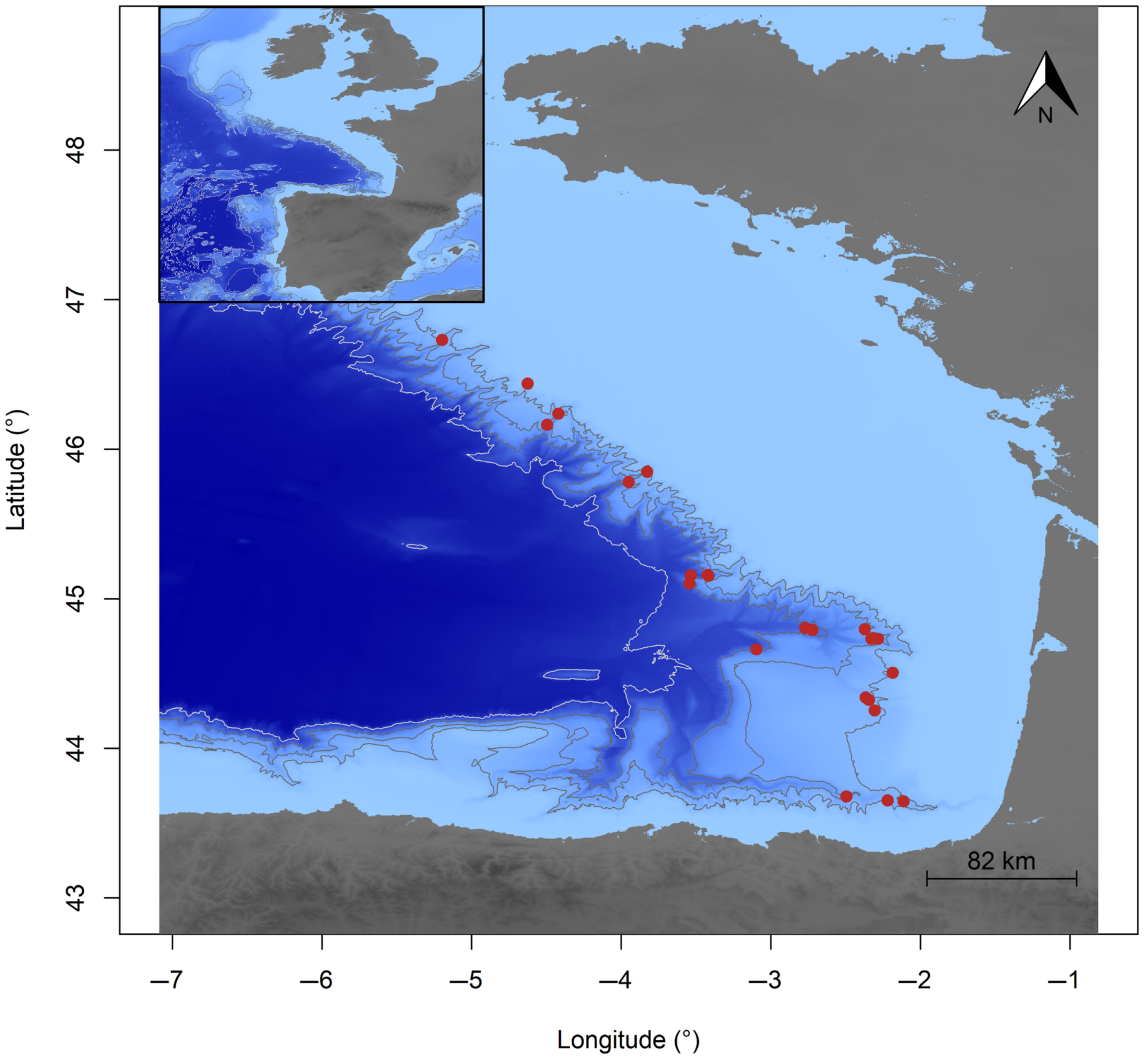
Trawl hauls' spatial position in the Bay of Biscay. The background blue colours represent the seabed depth (where lighter colours are shallower). The lines represent 1000, 2000, 3000 and 4000 m isobaths. The scale represents the number of kilometres for one degree of longitude (= 82 km).

Organisms were collected by epi‐ to bathypelagic trawling in canyons of the Bay of Biscay continental slope (North‐East Atlantic) during EVHOE scientific cruises (‘Evaluation Halieutique de l'Ouest de l'Europe’; https://doi.org/10.18142/8) that took place in autumn between 2002 and 2021. Trawls were conducted at night between 25 and 2000 m depth at 25 stations (Figure 1). The trawl net was 192 m long with a headline of 76 m and a foot rope of 70 m. The average vertical mean mouth opening was about 24 m and the horizontal opening of about 58 m. The mesh size gradually decreased from a very large 8 m (stretched mesh) at the mouth to 20 mm (stretched mesh) in the cod‐end. To allow the capture of very small specimens, the trawl was also equipped with a 7.5 m long sock with a 12 mm mesh size. Each trawl was performed at a specifically chosen immersion depth, meaning that only one depth was sampled at each station. The choice of trawl depth was determined by the depth of the scattering layer, with the additional goal of encompassing a broad depth range across the dataset. Consequently, some trawls were conducted significantly above or below the deep scattering layer. Once the trawl reached the selected depth it was towed horizontally (i.e. constant immersion depth) for 1 h at 4 kn. In addition, trawling was always carried out in complete darkness. The aim was to check whether the size distribution of a species remains consistent throughout the water column, particularly during the night feeding period when most species are active feeders (Eduardo et al., 2021; Kinzer & Schulz, 1985). Night sampling therefore presented optimal conditions for examining ontogenic movements.

### 2.2 Datasets

Two different datasets were used to study ontogenetic changes in deep‐sea pelagic fish species from the Bay of Biscay. The trawling (https://doi.org/10.4857/PRO/QE2VWQ) dataset included all data collected by trawls (i.e. number of individuals per species per sampling depth and total body length of each individual; *n* = 4165). To study the trophic aspects of ontogeny (see methodology below), muscle sampling was performed on 12 species of the trawling dataset to access the δ^15^N values of individuals (*n* = 682). This constituted the isotopic (https://doi.org/10.48579/PRO/O5QHTE) dataset. The size measured for the individuals sampled for the isotopic dataset was the standard length. As a result, size measurements differed between the two datasets. Total length was used for all catches made on board, as this is the usual measurement on fishing campaigns. However, the measurement used for individuals processed in the laboratory for analysis of δ^15^N values was standard length, as mesopelagic fish often have damaged tails. Based on repeated measurements of the species studied in the laboratory, we were able to compare the two measurements between our datasets. The conversions are available in the R code supplied. The size distribution of the individuals composing the species included in the isotopic dataset was representative of the size distribution observed in the trawling dataset (see Figure S1).

### 2.3 Nitrogen stable isotope analysis

A total of 682 muscle samples belonging to 12 of the most abundant species (seven migratory and five non‐migratory were collected) (Table 2). For each individual, the standard length (cm) was measured on board and a small piece of muscle was collected and frozen at −20°C. To have sufficient material for stable nitrogen isotope analysis, the muscles of the smallest individuals were pooled. Within each of these pools, the individuals were of equivalent size and were sampled at the same depths. At the laboratory, muscle samples were freeze‐dried (72 h). To reduce the samples to a fine powder, samples containing a single individual were manually homogenised, while samples containing a pool of individuals were homogenised using a ball mill (MM400 Retsch®) with zirconium oxide‐coated bowls and balls. A fraction of this powder (0.50 ± 0.05 mg dry mass) was weighed in tin cups. Analyses were then performed with an isotope ratio mass spectrometer (Delta V Advantage with a Conflo IV interface, Thermo Scientific) coupled to an elemental analyser (Flash EA, 2000; Thermo Scientific). Results are presented in the usual δ notation relative to the deviation from an international standard (atmospheric nitrogen, for δ^15^N values), in parts per thousand (‰). Based on repeated measurements of USGS‐61 and USGS‐62 used as laboratory internal standards, the experimental analytical precision was <0.15‰.

The isotopic dataset included individuals sampled from different years (i.e. between 2007 and 2021), which could have affected our data. However, more than 90% of the muscle samples were collected between 2019 and 2021 and nearly 75% in 2021, which reduces the potential inter‐annual effect. An analysis was also performed using only the years 2019 and 2021 to test the temporal variability and no effect changing our conclusions has been detected. At the community level, the δ^15^N‐size relationship remained the same (significant but weak relationship, *R* ^2^ < .01, *p* = .026). At the specific level, when the number of samples was sufficient, recalculation of the δ^15^N‐size relationships showed that the relationships remained consistent. We have therefore used all the data (from 2007 to 2021) for all the analyses. In addition, as sampling was always carried out during the same season (in autumn, at the end of October), potential seasonal variability bias was also limited. We hypothesise that changes in size and depth have a much greater influence on the δ^15^N values than does the temporal aspect. Details on the sampling by year for each species are presented in Figure S2. In addition, our sampling covered stations located at different latitudes within the bay, and this variability in the isotopic baseline could potentially affect our results. It is indeed difficult to disentangle the effects of depth, latitude and individual size on δ^15^N values. However, it is worth noting that all our sampling stations were situated in canyons at relatively consistent distances from the plateau, ranging from 9 to 32 km from the 200 m isobath, and the latitudinal range is limited (~3°). This geographical consistency minimises the potential variations in the baseline. Moreover, in the Bay of Biscay, differences in δ^15^N values were observed between the northern and southern regions for coastal species, primarily driven by variations in river discharge, but not for oceanic species (Chouvelon et al., 2012). To test this factor in our dataset, we used a variance partitioning analysis of δ^15^N values in relation to depth and latitude across all data. However, we did not observe any significant effect (*p*‐value depth = .122, *p*‐value latitude = .619).

### 2.4 Relationships between size distribution and depth

The different depth layers were defined as follows: the epipelagic zone between 25 and 175 m, the upper mesopelagic zone between 175 and 700 m, the lower mesopelagic zone between 700 and 1000 m and the bathypelagic zone below 1000 m. This division corresponds to the one used in the literature (Sutton, 2013) and is congruent with the depth structuration observed in the canyons of the Bay of Biscay (Loutrage et al., 2023). To study the changes in size distribution with depth, the trawling dataset was used. At both community and specific levels, a linear model was performed, the sampling depth corresponding to a continuous explanatory variable. Results were considered significant when the *p*‐value was ≤.05.

### 2.5 Relationships between δ^15^N values and size

The relationship between δ^15^N values and individual size was explored with the isotopic dataset at both community and species levels. In the case of pooled samples for nitrogen isotope analysis, the size data are the mean size of all pooled individuals. Linear models were employed for both species‐level and community‐level analyses, ensuring that the models adhered to the underlying assumptions of linear regression. Coefficients of variation were also calculated to assess the dispersion of values.

### 2.6 Variance partitioning

Variance partitioning was used to calculate the variance explained by the different variables included in a model (Borcard et al., 1992; Legendre & Legendre, 2012). This is done by developing a set of partial models (in a multivariate or univariate framework) created using a subset of predictor variables. Here, the objective was to test to what extent the individual size and the sampling depth influence the δ^15^N values at the specific level. Due to the restricted depth range at which *Aphanopus carbo* and *Stomias boa* were captured (≤100 m), the variance partitioning was not performed on these two species. The model results are composed of the proportion of δ^15^N values influenced by size and depth separately, and a third fraction representing the shared fraction of variation explained when both variables are included in the model. An ANOVA‐type permutation test was performed for each model to test the significance of the influence of each variable (depth and size) on δ^15^N values. Since the third fraction is deduced from the sum of variances, it cannot be tested statistically. The R package *vegan* was used to perform the tests (Oksanen et al., 2022). All the graphics were performed with the *ggplot2* R package and all statistical analyses were performed in the R environment version 4.3 (R Core Team, 2023; Wickham et al., 2016).

## 3 RESULTS

### 3.1 Relationships between size distribution and depth

**FIGURE 2:**
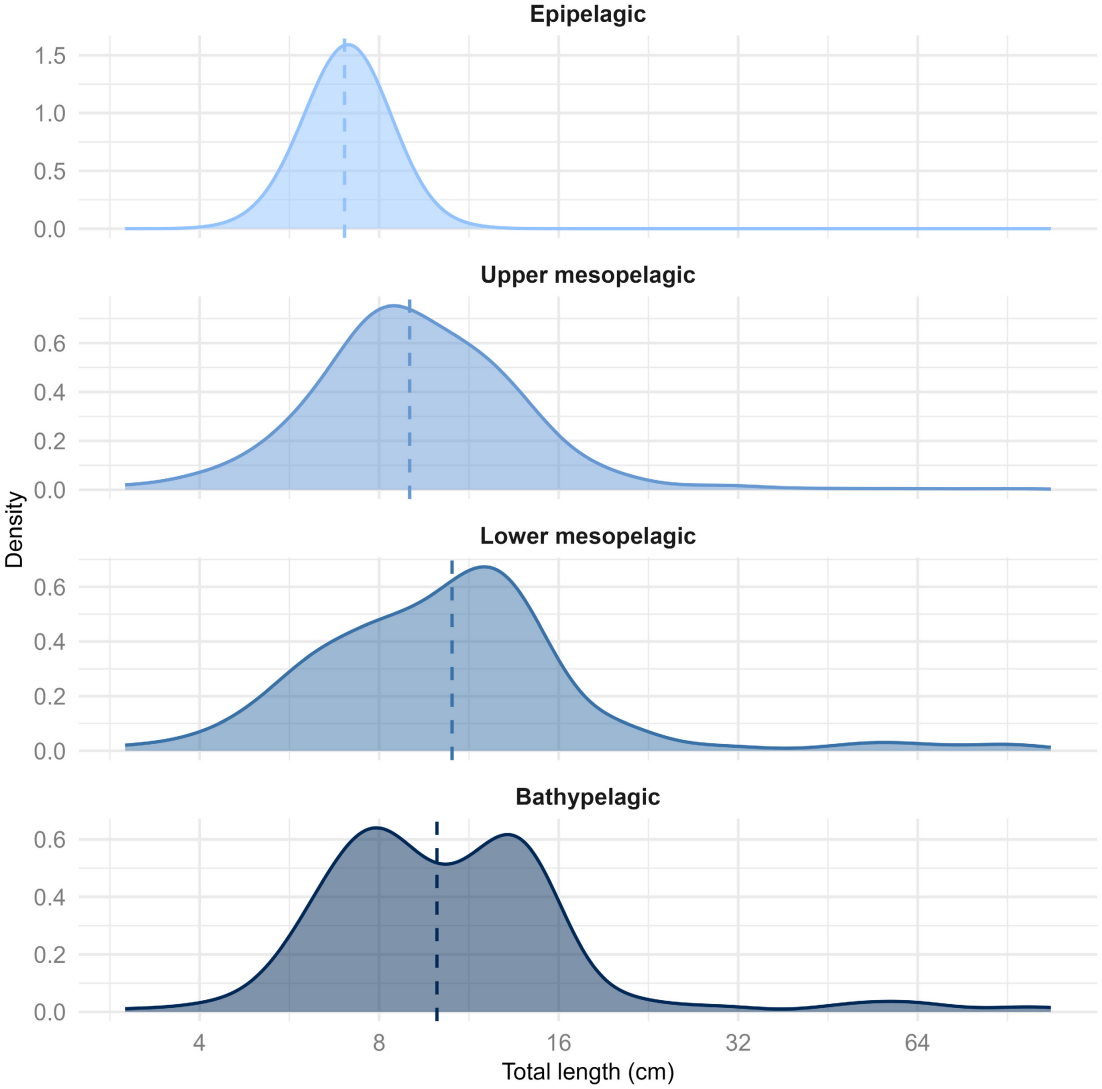
Size distribution (total length in cm) of individuals of the deep pelagic fish community according to the different depth layers (epipelagic: 25–175 m, upper‐mesopelagic: 175–700 m, lower‐mesopelagic: 700–1000 m and bathypelagic: ≥1000 m). The *x*‐axis is in log_2_ for clarity. The median size is indicated for each depth layer by a dashed line.

We observed a significant increase (*p*‐value < .001) in fish size (total length) with depth at the community level (Table 1). The median individual size increased consecutively between the epipelagic, upper mesopelagic and lower mesopelagic depth layers (median individual size equal to 7.0, 9.0 and 10.6 cm respectively; Figure 2). Median individual size then decreased slightly between the lower mesopelagic and bathypelagic layers, with a median individual size of 10 cm in the bathypelagic layer. Furthermore, while the first three depth layers had an unimodal distribution, the bathypelagic layer presented a bimodal distribution with a peak of around 8 cm and another around 13 cm.

**TABLE 1:** Results of the linear models at both community and species levels between size distribution and depth.

Family	Species		*N*	Slope	*R* ^2^	*p*‐value
Alepocephalidae	*Xenodermichthys copei* (Bluntsnout smooth‐head)	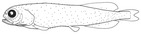	1070	1 × 10^−3^	.01	**<.001**
Myctophidae	*Lampanyctus crocodilus* (Jewel lanternfish)	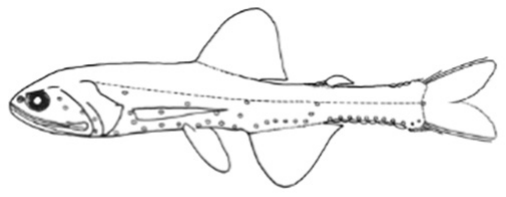	1200	2 × 10^−3^	.05	**<.001**
	*Lampanyctus macdonaldi* (Rakery beaconlamp)	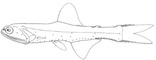	72	2 × 10^−4^	<.001	.605
	*Myctophum punctatum* (Spotted lanternfish)	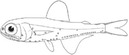	397	3 × 10^−4^	.01	**.040**
	*Notoscopelus kroyeri* (Lancet fish)	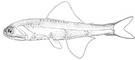	585	3 × 10^−5^	<.01	.912
Paralepididae	*Arctozenus risso* (Spotted barracudina)	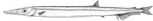	246	7 × 10^−4^	<.01	.226
Platytroctidae	*Searsia koefoedi* (Koefoed's searsid)	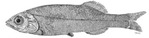	68	2 × 10^−4^	<.01	.863
Serrivomeridae	*Serrivomer beanii* (Stout sawpalate)	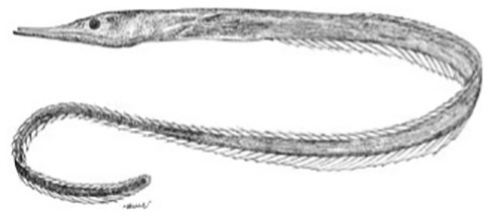	63	6 × 10^−4^	<.01	.866
Sternophidae	*Argyropelecus olfersii* (Olfer's Hatchetfish)	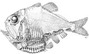	205	1 × 10^−4^	<.01	.706
Stomiidae	*Stomias boa* (Boa dragonfish)		63	2 × 10^−4^	<.01	.939
Trichiuridae	*Aphanopus carbo* (Black scabbardfish)		39	1 × 10^−2^	.06	.136
Zoarcidae	*Melanostigma atlanticum* (Atlantic soft pout)	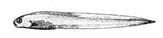	157	5 × 10^−3^	.15	**<.001**
Community	‐		4165	3 × 10^−3^	.01	**<.001**

*Note*: Significant relationships are shown in bold.

**FIGURE 3:**
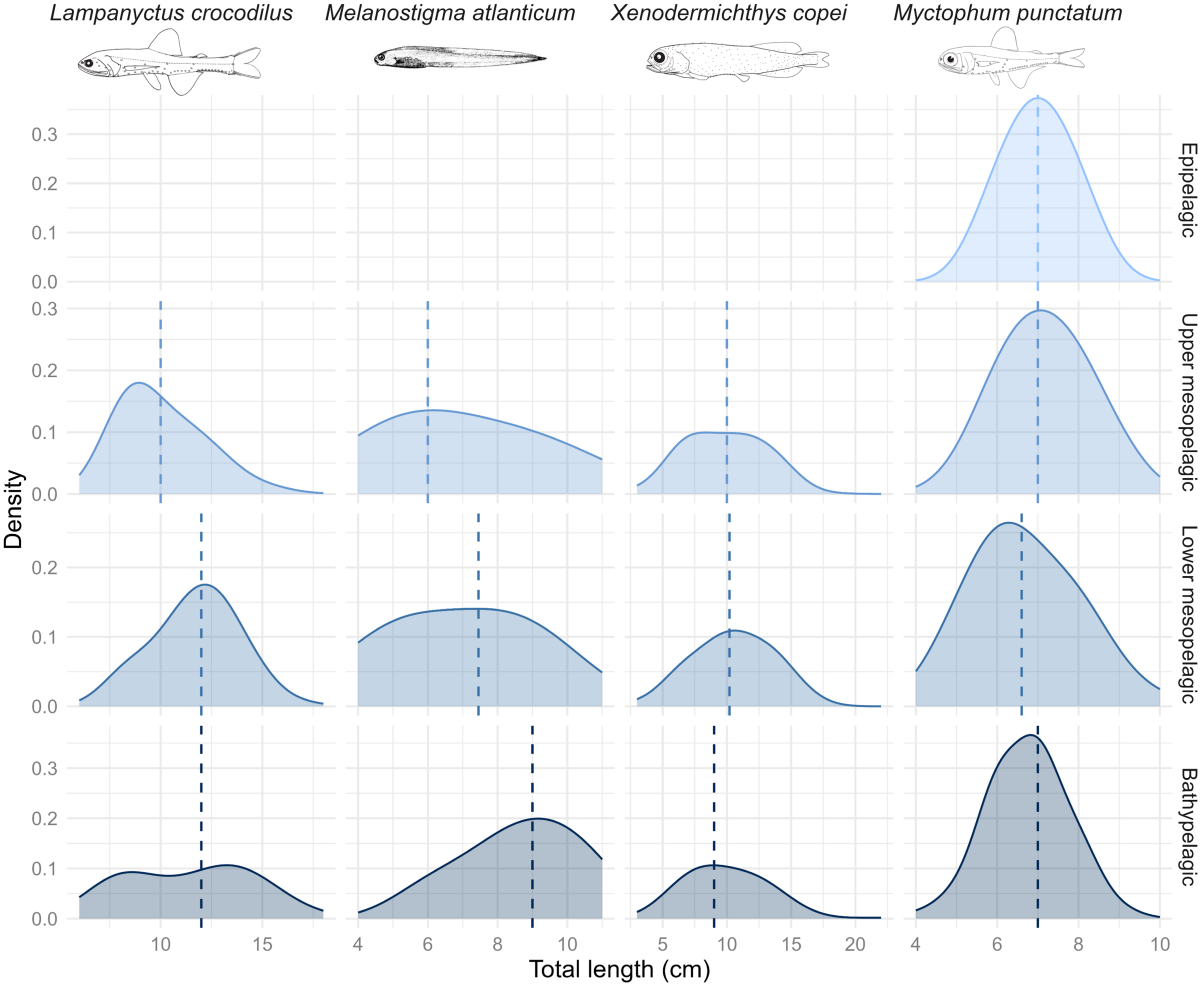
Size distribution (total length in cm) as a function of depth layer for the four species that showed significant relationships. The *x*‐axis is in log_2_ for clarity. The median size is indicated for each depth layer by a dashed line.

The relationship between size distribution and depth was also analysed at the species level (Table 1). Among the 12 species examined, only four exhibited a statistically significant linear relationship in the models. *Melanostigma atlanticum*, *L. crocodilus* and *Xenodermichthys copei* showed a significant increase in individual size with depth. *Myctophum punctatum* was the only species showing a significant decrease in the individual size with depth. *Lampanyctus crocodilus* showed an increase in individual size between the upper and lower mesopelagic layers, from a median size (total length) of 10.0–12.0 cm (Figure 3). This was followed by a stabilisation between the lower mesopelagic and bathypelagic layers with the same median size of 12.0 cm. *Melanostigma atlanticum* showed a continuous increase in the size of individuals with depth, with median individual sizes of 6.0, 7.45 and 9 cm respectively. *Xenodermichthys copei* showed a maximum median size in the lower mesopelagic layer (= 10.2 cm total length). Although *M. punctatum* showed a significant decrease in the size of its individuals with increasing depth (*p*‐value = .040), there appeared to be little variation between depth layers, with a median size of between 6.6 and 7.0 cm. Relationships for other species are available in Figure S3.

### 3.2 Relationships between δ^15^N values and size

δ^15^N values were determined for seven migratory and five non‐migratory or short migratory species, for a total of 682 individuals (= isotopic dataset). Mean δ^15^N values ranged from 9.49 ± 0.57‰ for *Serrivomer beanii* to 12.36 ± 0.33‰ for *A. carbo* (Table 2). For this dataset, mean values of standard length ranged from 6.3 ± 1.7 cm for *Argyropelecus olfersii* to 77.3 ± 10.8 cm for *A. carbo*, with *S. beanii* having the widest size range (45.0 cm between the minimum and the maximum length) and *Lampanyctus macdonaldi* the narrowest (3.3 cm).

**TABLE 2:** Number of samples for stable isotope analysis (*N*) and the total number of individuals (*n*) when some pools were made for the species, the minimum and maximum standard length of individuals (size, cm), sampling depth range (m) and mean ± standard deviation of δ^15^N values for each species.

Family	Species		*N* samples (*n* individuals)	Migratory pattern	Sampling depth (m)	Size range (cm)	Max size FishBase (cm)	δ^15^N (‰)
Alepocephalidae	*Xenodermichthys copei* (Bluntsnout smooth‐head)	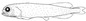	114 (126)	DVM	370–1335	5.6–9.5	31.0	9.83 ± 0.64
Myctophidae	*Lampanyctus crocodilus* (Jewel lanternfish)	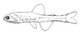	142 (154)	DVM	370–1600	6.5–14.8	30.0	10.46 ± 0.66
	*Lampanyctus macdonaldi* (Rakery beaconlamp)	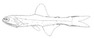	23	Probably no DVM^a^	1335–2000	11.5–14.8	16.0	11.54 ± 0.31
	*Myctophum punctatum* (Spotted lanternfish)	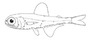	80 (95)	DVM	25–1335	5.0–9.0	11.0	9.99 ± 0.51
	*Notoscopelus kroyeri* (Lancet fish)	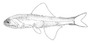	75 (181)	DVM	25–780	3.6–11.0	14.3	11.18 ± 0.24
Paralepididae	*Arctozenus risso* (Spotted barracudina)	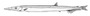	78 (89)	No DVM	370–780	11.0–20.5	30.0	10.53 ± 0.36
Platytroctidae	*Searsia koefoedi* (Koefoed's searsid)	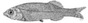	20 (22)	Probably no DVM^a^	750–1000	8.5–14.5	15.0	11.80 ± 0.57
Serrivomeridae	*Serrivomer beanii* (Stout sawpalate)	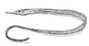	31 (37)	DVM	715–1335	26.5–75.5	78.0	9.49 ± 0.54
Sternophidae	*Argyropelecus olfersii* (Olfer's Hatchetfish)	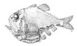	64 (70)	Short DVM	370–1335	3.3–10.0	9.0	10.18 ± 0.47
Stomiidae	*Stomias boa* (Boa dragonfish)	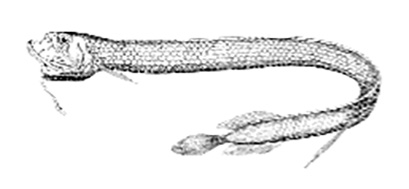	20 (30)	DVM	680–780	11.8–33.6	32.2	11.61 ± 0.71
Trichiuridae	*Aphanopus carbo* (Black scabbardfish)	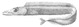	9	DVM	750–780	59.0–102.0	151.0	12.36 ± 0.33
Zoarcidae	*Melanostigma atlanticum* (Atlantic soft pout)	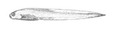	26 (34)	No DVM	730–1010	7.0–11.0	15.0	11.21 ± 0.50

*Note*: Migratory patterns are from www.fishbase.org, Lusher et al. (2016) and references therein.

Abbreviation: DVM, Diel vertical migration.

^a^ No DVM, at least towards epipelagic waters at night for feeding but possible migrations deeper into the bathypelagic zone (Moore et al., 2003; Novotny, 2018).

**FIGURE 4:**
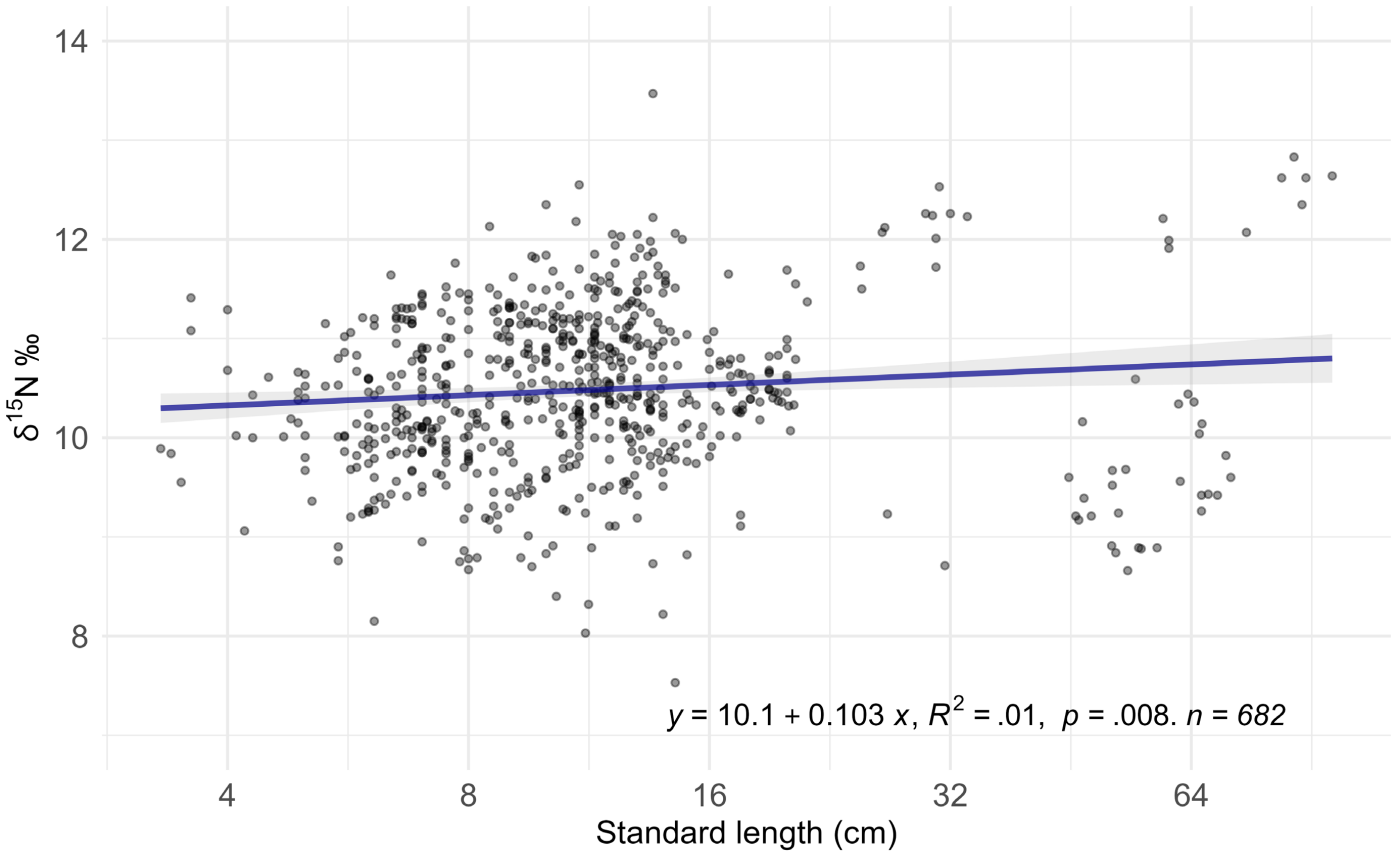
δ^15^N as a function of individual size (standard length, in cm) at the community level. The colour shades of the points correspond to their superposition. The *x*‐axis is in log_2_ for clarity.

The relationship between δ^15^N values and individual size was first investigated at the community level (Figure 4). The linear model results showed a significant increase in δ^15^N values with individual size. However, the *R* ^2^ was very low (= .01), indicating high variability in the values.

**FIGURE 5:**
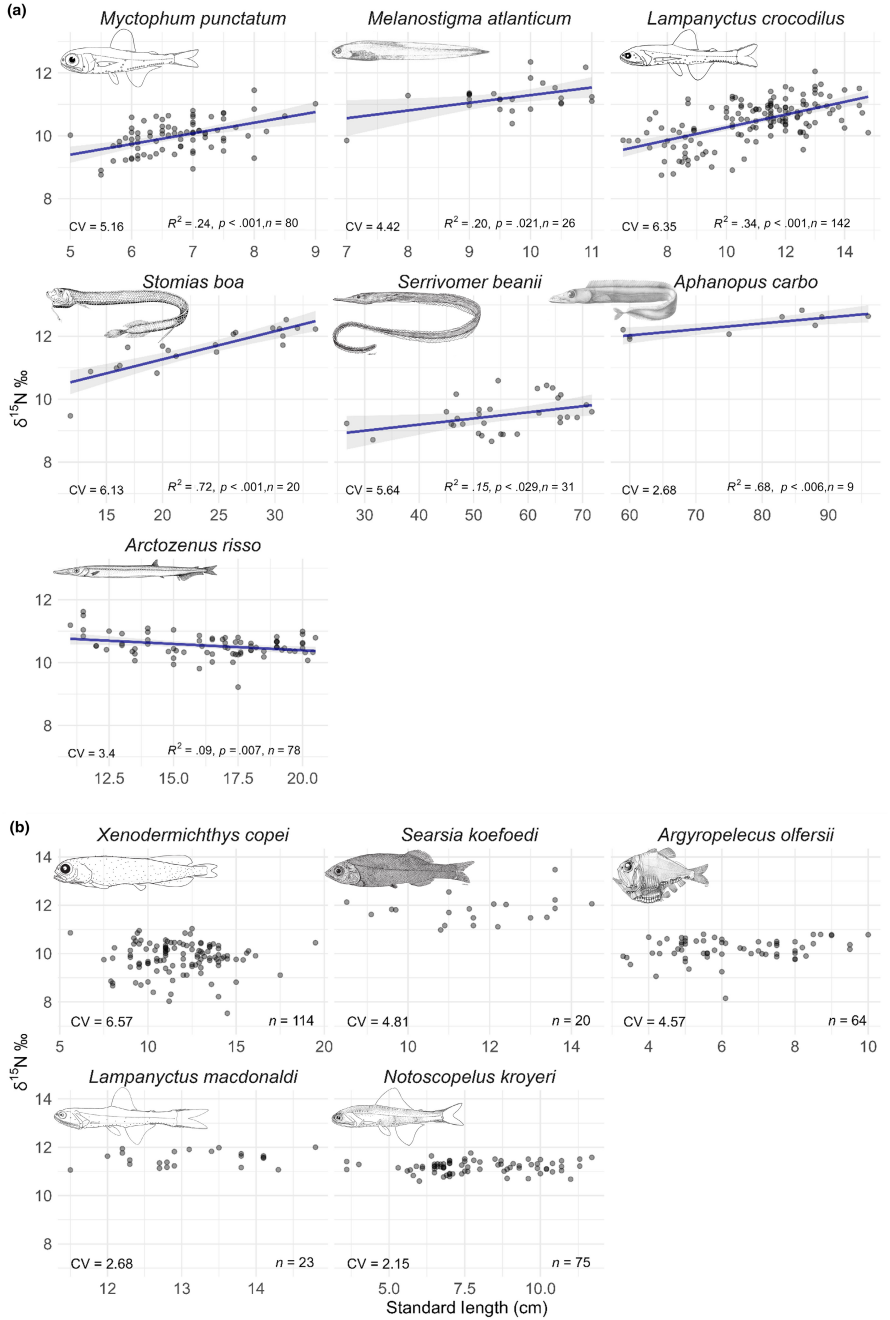
Relationships between δ^15^N values and individual size (standard length, in cm). (a) significant relationships and (b) non‐significant relationships. For the non‐significant relationships, the coefficient of variation (CV) is shown. The colour shades of the points correspond to their superposition.

The relationship between δ^15^N values and size was then investigated at the species level (Figure 5a). The results of the individual linear models showed that six species had a significant increase of δ^15^N values with increasing individual size: *M. punctatum*, *M. atlanticum*, *L. crocodilus*, *S. boa*, *S. beanii* and *A. carbo*. Only *Arctozenus risso* had a significant decrease of δ^15^N values with increasing size. In addition, differences in *R* ^2^ values were observed among species. In fact, despite the statistical significance of certain relationships, the low *R* ^2^ values may indicate that, from an ecological point of view, size explains little of the variation in isotopic values. *Stomias boa* had an *R* ^2^ of .72, indicating that the relationship between δ^15^N and size was accurately predicted by linear regression. In contrast, *A. risso* had a lower *R* ^2^ of .09, revealing that linear regression less accurately predicted the change in δ^15^N values of individuals with increasing body size for this species. The other five species had no significant relationship between the two variables (Figure 5b). The δ^15^N values were stable regardless of the size increase. However, the coefficient of variations allowed to distinguish species with low interindividual variability (e.g. *Notoscopelus kroyeri*) from species with high interindividual variability (*X. copei*).

### 3.3 Variance partitioning

**FIGURE 6:**
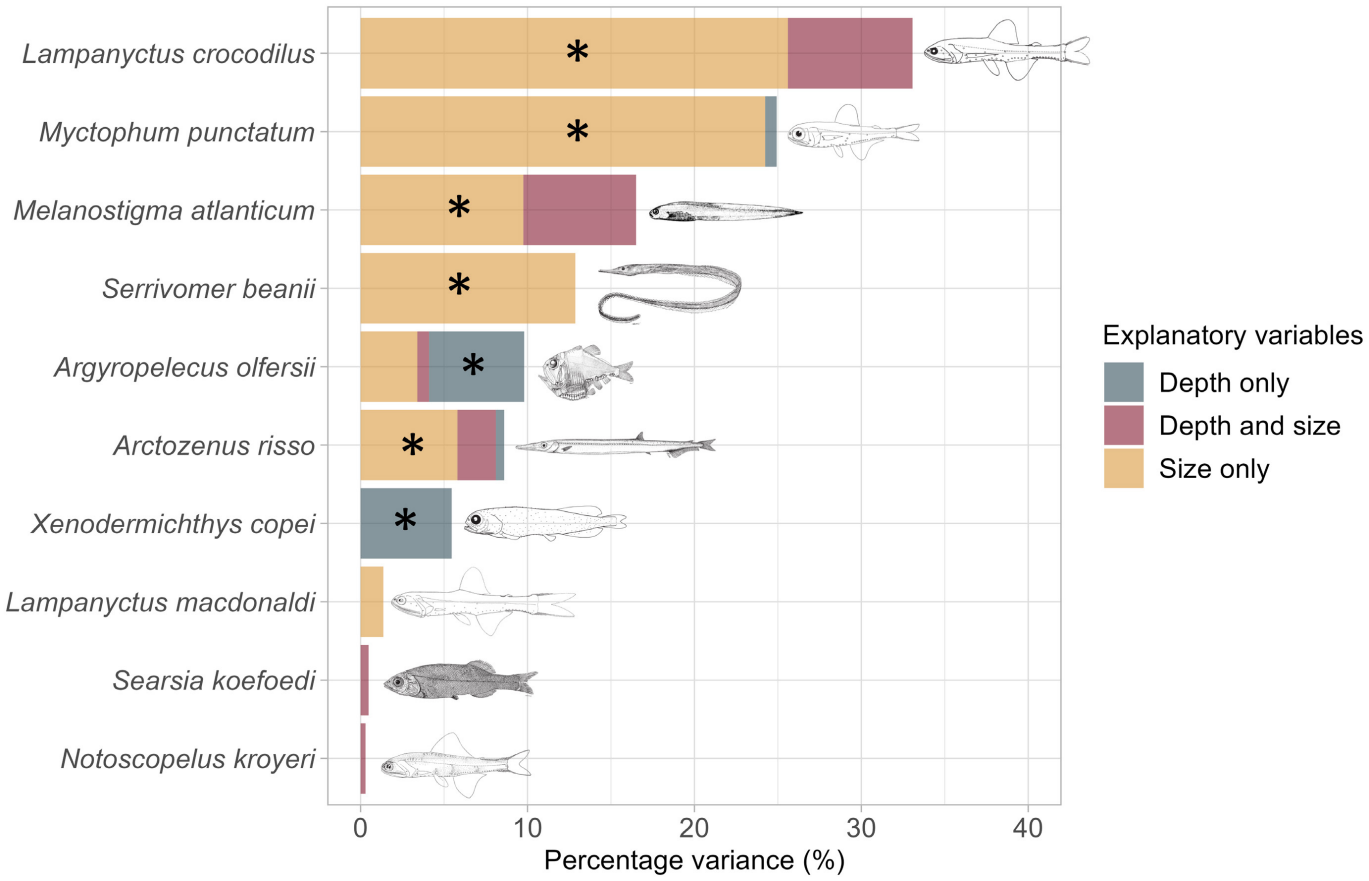
Proportions of variance in δ^15^N values explained by size and depth alone and by size and depth together. Asterisks indicate a significant influence of one or other of the variables tested. The unexplained variance has been omitted for graphical clarity and corresponds to the difference between one and the sum of the explained variance.

Results of the variation partitioning analyses showed that five species (the same as above) had their δ^15^N values significantly influenced by individual size (Figure 6 and Table 3, NB: *S. boa* and *A. carbo* not considered in these analyses due to small depth range). *L. crocodilus* had the highest proportion of variation in δ^15^N values explained by size (25.7%), followed by *M. punctatum* (24.7%), *M. atlanticum* (10.1%) and *S. beanii* (13.2%) and *A. risso* (5.8%). Alternatively, in *A. olfersii* and *X. copei*, variations in δ^15^N were significantly explained by depth, at a proportion of 5.7% and 5.5% respectively.

**TABLE 3:** Results of the variance partitioning analysis (ANOVA) for each species.

Species	Variable	Variance	*F* value	*p*‐value
*Xenodermichthys copei* (Bluntsnout smooth‐head)	Size	<0.001	0.058	.824
	Depth	0.026	7.48	**.009**
*Lampanyctus crocodilus* (Jewel lanternfish)	Size	0.115	54.5	**.001**
	Depth	<0.001	0.081	.771
*Lampanyctus macdonaldi* (Rakery beaconlamp)	Size	0.006	1.30	.252
	Depth	0.003	0.578	.477
*Myctophum punctatum* (Spotted lanternfish)	Size	0.067	26.2	**.001**
	Depth	0.004	1.75	.202
*Notoscopelus kroyeri* (Lancet fish)	Size	<0.001	0.320	.578
	Depth	<0.001	0.480	.500
*Arctozenus risso* (Spotted barracudina)	Size	0.009	5.78	**.019**
	Depth	0.002	1.43	.219
*Searsia koefoedi* (Koefoed's searsid)	Size	0.016	0.880	.380
	Depth	0.006	0.350	.578
*Serrivomer beanii* (Stout sawpalate)	Size	0.045	5.28	**.032**
	Depth	0.003	0.409	.510
*Argyropelecus olfersii* (Olfer's Hatchetfish)	Size	0.010	3.34	.072
	Depth	0.015	4.92	**.026**
*Melanostigma atlanticum* (Atlantic soft pout)	Size	0.032	3.81	**.044**
	Depth	<0.001	<0.001	.994

*Note*: Values in bold are those showing a significant influence of the variable (*p*‐value <.05).

### 3.4 Summary of relationships at specific and community levels

Based on the distinct relationships between size‐depth and δ^15^N‐size, various species patterns are described (Table 4). Specifically, two species (*L. crocodilus* and *M. atlanticum*) showed body size change in both their vertical distribution and their δ^15^N values. Larger individuals of these species were caught at greater depth and their δ^15^N values increased with individual size. Meanwhile, four other species showed a change in their δ^15^N values with body size but showed no variation in their vertical distribution (from trawling data): *A. risso*, *S. beanii*, *S. boa* and *A. carbo*. Among the species that did not show any significant change in their trophic ecology (as indicated by δ^15^N values) with increasing size, variations in the dispersion of δ^15^N values were observed. Some species such as *N. kroyeri* and *L. macdonaldi* had a restricted range of δ^15^N values whatever the size of individuals (i.e. variation coefficients = 2.15 and 2.68 respectively) whereas some species such as *X. copei* showed a high dispersion of δ^15^N values (CV = 6.57). Finally, three species showed no relationships among the variables tested: *N. kroyeri*, *L. macdonaldi* and *Searsia koefoedi*. However, for *L. macdonaldi*, a particularly low size range was sampled for isotopic analysis (= 3 cm between the minimum and the maximum individual size). At the community level, an increase in individual size with depth was observed, as well as an increase in δ^15^N values with increasing size (Table 4).

**TABLE 4:** Summary of the relationships investigated for each species and the community considered as a whole.

Species	Family	Mechanism driving ontogenetic shift	Relationships	Description
*Lampanyctus crocodilus*	Myctophidae	Trophic & and habitat shifts with body size 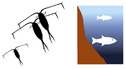	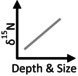	Species showing shifts in both their vertical distribution (largest individuals at greater depth) and their trophic ecology (increase of δ^15^N values with the size of the individuals) with body size.
*Melanostigma atlanticum*	Zoarcidae			
*Myctophum punctatum*	Myctophidae	Trophic shift with body size 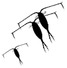	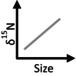	Species showing only a shift in their trophic ecology with body size (influence of individual size on δ^15^N values, but no effect of depth whenever it could be tested).
*Arctozenus risso*	Paralepididae			
*Serrivomer beanii*	Serrivomeridae			
*Stomias boa* ^a^	Stomiidae			
*Aphanopus carbo* ^a^	Trichiuridae			
*Argyropelecus olfersii*	Sternoptychidae	Depth‐driven increase in δ^15^N values	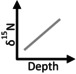	Species showing an increase of their δ^15^N values with depth (but no effect of size). *X. copei* also showed a shift in the vertical distribution of individuals (the smallest individuals were not found in the deepest stations).
*Xenodermichthys copei*	Alepocephalidae			
*Notoscopelus kroyeri*	Myctophidae	No shift	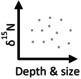	Species with no observed shifts in relation with body size (no effect of depth on δ^15^N values). Moreover, *N. kroyeri* and *L. macdonaldi* had a low CV of their δ^15^N values (CV < 3) while *S. koefoedi* had a wider dispersion of δ^15^N values (CV = 4.81).
*Lampanyctus macdonaldi*	Myctophidae			
*Searsia koefoedi*	Platytroctidae			
Community	‐	Trophic & and habitat shifts with body size 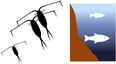	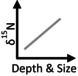	The whole community showed an increase in individual sizes with depth. A weak but significant relationship was also found between δ^15^N values and individual size.

Abbreviation: CV, Coefficient of variation.

^a^ Only the δ^15^N‐size relationship was tested for *A. carbo* and *S. boa* due to too low depth ranges sampled for these two species (test with linear models only, not through variance partitioning analyses).

## 4 DISCUSSION

The present study confirms, for the Bay of Biscay, the global trend towards observing larger fish at greater depth for deep pelagic fish communities, as described in other systems (Auster et al., 1992; Badcock & Merrett, 1976; Gartner et al., 1997; Stefanescu & Cartes, 1992; Willis & Pearcy, 1980). The presence or not of ontogenetic shifts and their associated main drivers (trophic and/or habitat) were described: (i) species undergoing both body size shifts in vertical habitat use (as inferred from trawling data) and in their trophic ecology (as inferred by δ^15^N values): *L. crocodilus* and *M. atlanticum*; (ii) species showing only a body size shift in their trophic ecology (i.e. the significant influence of size on δ^15^N values): *M. punctatum*, *A. risso*, *S. beanii*, *S. boa* and *A. carbo*; (iii) species for which only depth influences their δ^15^N values (*X. copei* and *A. olfersii*), although *X. copei* also presented an ontogenetic shift in habitat use from trawling data (with the smallest individuals not found in the deepest stations) and (iv) species showing no ontogenetic shift: *L. macdonaldi*, *S. koefoedi* and *N. kroyeri*.

When investigating ontogenetic shifts in habitat use and trophic ecology within deep pelagic fish communities, several key aspects must be considered. One primary limitation is the restricted depth or size range covered by the isotopic dataset for certain species. In fact, not all the individuals in the trawl dataset could be sampled for the analysis of stable isotopes of nitrogen. In particular, species like *A. carbo* and *S. boa*, which had depth ranges of 685 and 1630 m, respectively, in the trawl dataset, had their sampled individuals collected in a depth range of less than 100 m. Alternatively, *L. macdonaldi* was sampled over a small size range (≈3 cm), which may potentially explain the lack of significant relationships found with individual size for this species. Given that the isotopic sampling spanned 14 years, potential temporal bias in δ^15^N values cannot be excluded. However, the same analyses performed exclusively on data from 2019 to 2021 yielded similar results for the δ^15^N‐size relationship at the community and specific levels, indicating that the temporal variability of δ^15^N values remains relatively low in our dataset (i.e. linear relationships remain unchanged). Additionally, it is important to note that all samples were collected during the autumn season. In addition, the influence of organism physiology on δ^15^N values cannot be discounted, especially for fast‐growing species (Vander Zanden et al., 2015). While this parameter was not tested, the lack of significant size changes among several species with varying δ^15^N values suggests that growth does not affect these values. We assumed that the size and depth parameters have a greater impact on these values.

### 4.1 Community level

A significant increase in the size of individuals with depth was observed at the community level. This pattern has already been observed for migratory species, with older stages generally found at greater depths than younger ones, as individuals that may have reduced their migratory range or stopped migrating with age (Auster et al., 1992; Badcock & Merrett, 1976; Clarke & Wagner, 1976; Gartner et al., 1997; Lancraft et al., 1988; Nafpaktitis, 1977; Stefanescu & Cartes, 1992; Willis & Pearcy, 1980). This general trend in the deep pelagic realm may be a consequence of the trade‐off between foraging and predation. To satisfy their energetic needs, juveniles and adults of some species migrate to the epipelagic layer to feed at night. Alternatively, at the senescent stage, some species undergo a reduction in swim bladder size as they age (e.g. *L. crocodilus*), so that the energetic cost of migration may be greater than the benefit provided. Some of these species, therefore, adopt a benthopelagic behaviour which allows them to reduce the energy expended on foraging by taking advantage of the higher concentrations of zooplankton in the benthopelagic zone (Angel & Baker, 1982; Vinogradov, 2005).

At the community level, a slight but significant increase in δ^15^N values with individual size was also found. However, this relationship was very weak (*R* ^2^ = .01). Indeed, it has previously been shown that within fish communities, the increase in δ^15^N values as a function of individual size was more strongly linked to ontogenetic changes than to the fact that the largest species in the community fed on higher trophic‐level prey (Jennings et al., 2002; Stowasser et al., 2012). There are several possible causes: omnivory, large predators feeding on smaller prey, large pelagic suspension feeders feeding on small suspended particulate organic matter and morphological adaptations of small predators to feed on larger prey (Bode et al., 2007; Jacob, 2005; Jennings, 2005). In particular, in our study, *S. beanii* was the second largest species sampled (= 55 cm) but had the lowest mean δ^15^N values (= 9.5‰). This could be partly explained by their serpentine morphology, with a large individual size that is not proportionally reflected in the size of the mouth opening, limiting their ability to capture large prey compared to other species with similar individual sizes. Indeed, our study includes a wide variety of morphology, including lanternfish, hatchetfish, dragonfish and eelfish. This diversity in shape is bound to lead to large differences in feeding strategies. Consequently, body size may not be the best measure to infer the trophic ecology of these species, although it is often correlated with several other measures. The analysis was conducted using individual body mass, and the correlation exhibited identical patterns (*R* ^2^ = .04, *p* > .001). For example, size or shape of the mouth opening may be more relevant to study this relationship at the community level (Villéger et al., 2017).

### 4.2 Trophic‐driven ontogenetic shift

Intraspecific shift in fish trophic ecology with body size (as inferred from δ^15^N values variation with size) is generally a consequence of the ability of fish to catch larger prey. This ability is proportional to mouth size, which in turn is proportional to body size, allowing species to feed on larger prey (Dunic & Baum, 2017). Such a pattern has already been observed in mesopelagic fish species (Gartner et al., 1997). In the present study, half of the studied species showed a significant change in their δ^15^N values with the increasing size of individuals. Of these species, four had their δ^15^N values influenced solely by the size of individuals: *M. punctatum*, *S. beanii*, *S. boa* and *A. carbo*. However, in the case of *A. carbo* and *S. boa*, the depth range sampled for these species was too small to test the other relationships. *M. punctatum* was previously described as a generalist feeder in the Mediterranean Sea with a mixed diet during all stages of its development, except small individuals that seem to feed exclusively on copepods (Scotto di Carlo et al., 1982). As they grow, individuals become more efficient predators and begin to select larger, more nutritious prey (Bernal et al., 2015). In another study in the Northern Atlantic, *A. carbo* also showed an increase in δ^15^N values with individual size, confirming the probable ontogenetic diet or trophic level shift for this species (Farias et al., 2014). Its diet would shift from pelagic zooplankton to bathypelagic prey, reflecting an improvement in its predatory ability (Farias et al., 2014). To our knowledge, this is the first time that a shift in trophic ecology with body size is reported for *S. beanii* and *S. boa*. The diet of *S. beanii* is generally described as being composed of crustaceans and small fish, whereas that of *S. boa* is composed of crustaceans and mesopelagic fish, so our results suggest that the proportions may vary with individual size (Whitehead et al., 1984). Finally, among the studied species, only *A. risso* has undergone a significant but weak decrease in δ^15^N values with individual size. This trend has already been found for two species of the southern Kerguelen mesopelagic community belonging to the families Platytroctidae and Myctophidae (Woods et al., 2019). A significant decrease in δ^15^N values was also found in a small pelagic neritic fish species, the European sardine (*Sardina pilchardus*). This reduction was attributed to the greater efficiency of large sardines in capturing phytoplankton, which is less enriched in ^15^N than zooplankton prey (Bode et al., 2003, 2004, 2006). In addition, several species belonging to the Paralepididae family (which includes *A. risso*) have shown tooth loss in adult specimens and a recent study in the western Atlantic also found this pattern for *A. risso* (Devine & Van Guelpen, 2021; Ho & Duhamel, 2019). This tooth loss may lead to dietary changes in this species, partially explaining the negative relationship between δ^15^N values and size found in our results.

### 4.3 Trophic and habitat‐driven ontogenetic shifts

*Lampanyctus crocodilus* and *M. atlanticum* showed, in addition to an increase in δ^15^N values with individual size, an increase in individual size with depth. In the Mediterranean Sea, *L. crocodilus* has already been shown to make a change in its diet in relation to its changes in migratory activity (Fanelli et al., 2014; Stefanescu & Cartes, 1992). *L. crocodilus* has a diet dominated by epipelagic crustaceans in its pelagic phase and its migratory activity decreases or even stops when it reaches the senescent stage. It then adopts a benthopelagic behaviour and feeds on fish at greater depth (Bernal et al., 2023; Stefanescu & Cartes, 1992; Valls et al., 2014). Our present results, therefore, suggest that this behaviour may also occur in the Bay of Biscay. As for *M. atlanticum*, it has a particular mode of reproduction. This species adopts a benthic behaviour during the spawning period and the fertilisation of its eggs takes place in burrows located under the surface of the sea bed (Dallarés et al., 2021; Silverberg et al., 1987). This specific reproductive behaviour may explain our results that the largest, and thus reproductive individuals are found at greater depth. From a trophic perspective, it has been shown in the Mediterranean Sea that the diet of *M. atlanticum* consists almost exclusively of pelagic prey (Dallarés et al., 2021). Larger individuals may have the ability to capture larger prey, which can also explain the relationship between δ^15^N values and individual size found for this species. These two species (*L. crocodilus* and *M. atlanticum*), in addition to having these two significant relationships (larger individuals are found deeper and have higher δ^15^N values), presented the highest percentages of the variance in δ^15^N values explained by both size and depth (i.e. the red portion, >7%) in the partition models. This part reflects the proportion of the model that cannot distinguish the effect of depth and size on δ^15^N values. The benthopelagic behaviour of these species in the adult stage may also partly explain this influence, as δ^15^N values (including those of POM at the base of food webs) are higher at greater depth and particularly in the benthic domain (Bergmann et al., 2009; Richards et al., 2020; Trueman et al., 2014).

### 4.4 Depth‐driven increase in δ^15^N values

Two species, *X. copei*, and *A. olfersii* had their δ^15^N values significantly influenced only by depth (and not by individual size). In addition, in the case of *X. copei* the smallest individuals were not found at greater depth. Like *M. atlanticum*, *X. copei* was previously reported to spawn demersally in the North Atlantic, with individuals in pelagic trawls that were juveniles and larger fish that were caught in the deeper stations near the bottom (Mauchline & Gordon, 1983). As the sampling in our study was carried out during the spawning season of this species (October–November), many spawning individuals were observed. The capture of large spawning individuals of *X. copei* at depth suggests that spawning of this species also occur on the slope area at that period in the Bay of Biscay. Although *X. copei* did not show a significant relationship with size, it stood out for its wide dispersion of values δ^15^N values (CV = 6.57). In the North Atlantic, pelagic individuals of this species have a diet limited mainly to copepods and ostracods, while benthic individuals show a wider variety of food, maybe explaining in part this high variability in δ^15^N values (Mauchline & Gordon, 1983). In addition, it has been observed that both immature and larger individuals may graze on inactive prey or debris such as small copepods, potentially decoupling the relationship between size and δ^15^N values (Roe et al., 1984). In addition, the δ^15^N values of *X. copei* were significantly influenced by depth. This may be a consequence of the reproductive individuals residing at depth for this period and therefore integrating the enrichment of the δ^15^N values at depth (Choy et al., 2015; Gloeckler et al., 2018; Richards et al., 2020; Romero‐Romero et al., 2019). As for *A. olfersii*, it is a short‐migratory species described to feed on crustaceans and small fish (Muus et al., 1999). It has already been shown that non‐migratory species such as *A. olfersii* integrate changes in zooplankton δ^15^N with depth (Hannides et al., 2013; Koppelmann et al., 2009). Indeed, individuals located deeper are more dependent on the food web based on bacterially degraded organic particles and thus enriched in δ^15^N than individuals located less deep (Choy et al., 2015; Gloeckler et al., 2018; Richards et al., 2020; Romero‐Romero et al., 2019). This result was already observed for two non‐migratory species near the island of Hawaii: *Cyclothone pallida* and *Melanocetus johnsonii* (Romero‐Romero et al., 2019). Among the non‐migratory species in our study, *A. olfersii* was the species with the largest range of depth sampled (≈1000 m) which may explain the significant influence of depth on δ^15^N values for this species. For the other non‐migratory species (i.e. *L. macdonaldi*, *A. risso*, *S. koefoedi*), the depth range sampled was maybe too small to detect any influence of depth on the δ^15^N values.

### 4.5 No ontogenetic change and no influence of depth on δ^15^N values cases

Finally, five species showed no trophic‐driven change with body size: *X. copei*, *S. koefoedi*, *A. olfersii*, *L. macdonaldi* and *N. kroyeri*. Among these species, some showed high variability in δ^15^N values (*X. copei*, *S. koefoedi*, *A. olfersii*), while others had relatively constant values across their size range (*L. macdonaldi* and *N. kroyeri*). This result could potentially reflect differences in feeding strategies between species. Species with a high dispersion of δ^15^N values may have higher dietary plasticity, allowing them to feed on a wide variety of prey. Such a pattern has already been found for several small pelagic neritic species such as *Scomber scombrus* in the Iberian Peninsula (Bode et al., 2006). However, information on the diet of the Platytroctidae family is very scarce in the literature. *S. koefoedi* has been reported to have a diet composed largely of copepods, but also ostracods, chaetognaths and polychaetes, which could partly explain the large variability in δ^15^N values found during its ontogeny (Hopkins et al., 1996; Novotny, 2018). In contrast, species with low variability in δ^15^N values could have implemented an alternative strategy to the one classically observed, based on an increase in the size of the prey associated with an increase in mouth size. In this case, meeting energy requirements would be based on an increase in the quantity of resources ingested, made possible by the increase in mouth size, while maintaining the same type (size) of food resources rather than a higher energy content per larger prey ingested. The two Myctophidae species of this group, *N. kroyeri*, and *L. macdonaldi* showed weak variability in δ^15^N values with increasing body size. These two species have been reported to have a diet mainly composed of crustaceans (Coad & Reist, 2004; Gjøsæter, 1981). However, for the *L. macdonaldi* case, the restricted size range sampled can explain part of this absence of relation (= 3 cm). In addition, the smaller individuals sampled had a standard length of 11.5 cm, which is important considering the maximum size of 16 cm reported for this species. In the case of *N. kroyeri*, individuals' diets may not be size‐restricted like in the case of filter feeders, with a strategy of increasing the quantity of ingested food with size.

### 4.6 The Myctophidae case study

An important observation from our results is that the Myctophidae species studied here appeared to have significant differences in their feeding strategies during ontogeny. They were found in three of the four groups of species formed from the different relationships. First, *L. crocodilus* differs from the other species in that it undergoes changes in both its diet and depth distribution with increasing body size. As large adults have a reduced swim bladder, the energy gain associated with nocturnal migration to feed in productive surface waters may outweigh the costs, making feeding in the benthic boundary layer more cost‐effective (Fanelli et al., 2014). While both *M. punctatum* and *N. kroyeri* are known to migrate vertically at night in the epipelagic layer, they appeared to have adopted opposite feeding strategies, with *M. punctatum* appearing to change its diet (i.e. increasing δ^15^N values with size), whereas not only was this change not seen in *N. kroyeri*, but its δ^15^N values remained very stable across the species size gradient. Thus, by not shifting its diet towards larger or more energetic prey with increasing body size, *N. kroyeri* appears to have opted for an increase in food quantity rather than a change in quality. Finally, *L. macdonaldi* had the deepest distribution (i.e. median depth = 2000 m), which probably explains in part the lack of profitability for this species to move into the epipelagic layer to feed at night. *L. macdonaldi* also appeared not to have undergone any dietary changes during ontogeny, although this remains to be confirmed with a wider sampling across the size range of the species. All these differences in the ontogenetic foraging strategies of these phylogenetically related species may be partially explained by differences in morphological traits. Indeed, differences in morphological traits can lead to differences in swimming ability, prey capture, detection ability visual acuity, etc (Villéger et al., 2017). Differences in feeding strategies and depth distribution in relation to body size suggest divergence within this highly diverse family to avoid competition.

## 5 CONCLUSION

Overall, we identified significant variations in foraging trade‐offs related to ontogenetic changes in the community. These variations could influence the functional roles played by the species within the ecosystem, highlighting the need for further incorporation into future research (Nakazawa, 2015). Network‐based approaches have demonstrated that the role of fish is stage‐specific in terms of their functionality, with significant impacts on energy pathways, food web structure and dynamics (Miller & Rudolf, 2011; Nakazawa, 2015; Ramos‐Jiliberto et al., 2011; Woodward et al., 2005). Therefore, characterising these changes for the 12 relatively unknown species examined in this study constitutes a crucial initial step towards a more profound comprehension of the trophodynamic functioning of deep pelagic food webs.

## AUTHOR CONTRIBUTIONS

**Liz Loutrage:** Conceptualization (equal); formal analysis (equal); investigation (equal); methodology (equal); validation (equal); visualization (equal); writing – original draft (lead). **Anik Brind'Amour:** Conceptualization (equal); formal analysis (equal); investigation (equal); methodology (equal); supervision (equal); validation (equal); visualization (equal); writing – original draft (equal). **Tiphaine Chouvelon:** Conceptualization (equal); formal analysis (supporting); investigation (equal); methodology (equal); supervision (supporting); validation (equal); visualization (equal); writing – original draft (equal). **Jérôme Spitz:** Conceptualization (equal); formal analysis (supporting); funding acquisition (lead); investigation (equal); methodology (equal); supervision (equal); validation (equal); visualization (equal); writing – original draft (equal).

## CONFLICT OF INTEREST STATEMENT

The authors declare no conflicts of interest.

## Supporting information


Figure S1

Figure S2

Figure S3

## Data Availability

The raw data supporting the conclusions of this article are publicly available through the data.InDoRES platform. The isotopic dataset is available at https://doi.org/10.48579/PRO/O5QHTE and the trawling dataset at https://doi.org/10.48579/PRO/QE2VWQ. The code to reproduce the full analysis is available on GitHub (https://github.com/lizloutrage/ontogeny_deep_pelagic_fish).
