# Supplementary figures and images for: Ontogenetic shift or not? Different foraging trade‐offs within the meso‐ to bathypelagic fish community
Source: Ecol Evol. 2024 Mar 20;14(3):e11129. doi: 10.1002/ece3.11129 (PMC10954512; doi:10.1002/ece3.11129)

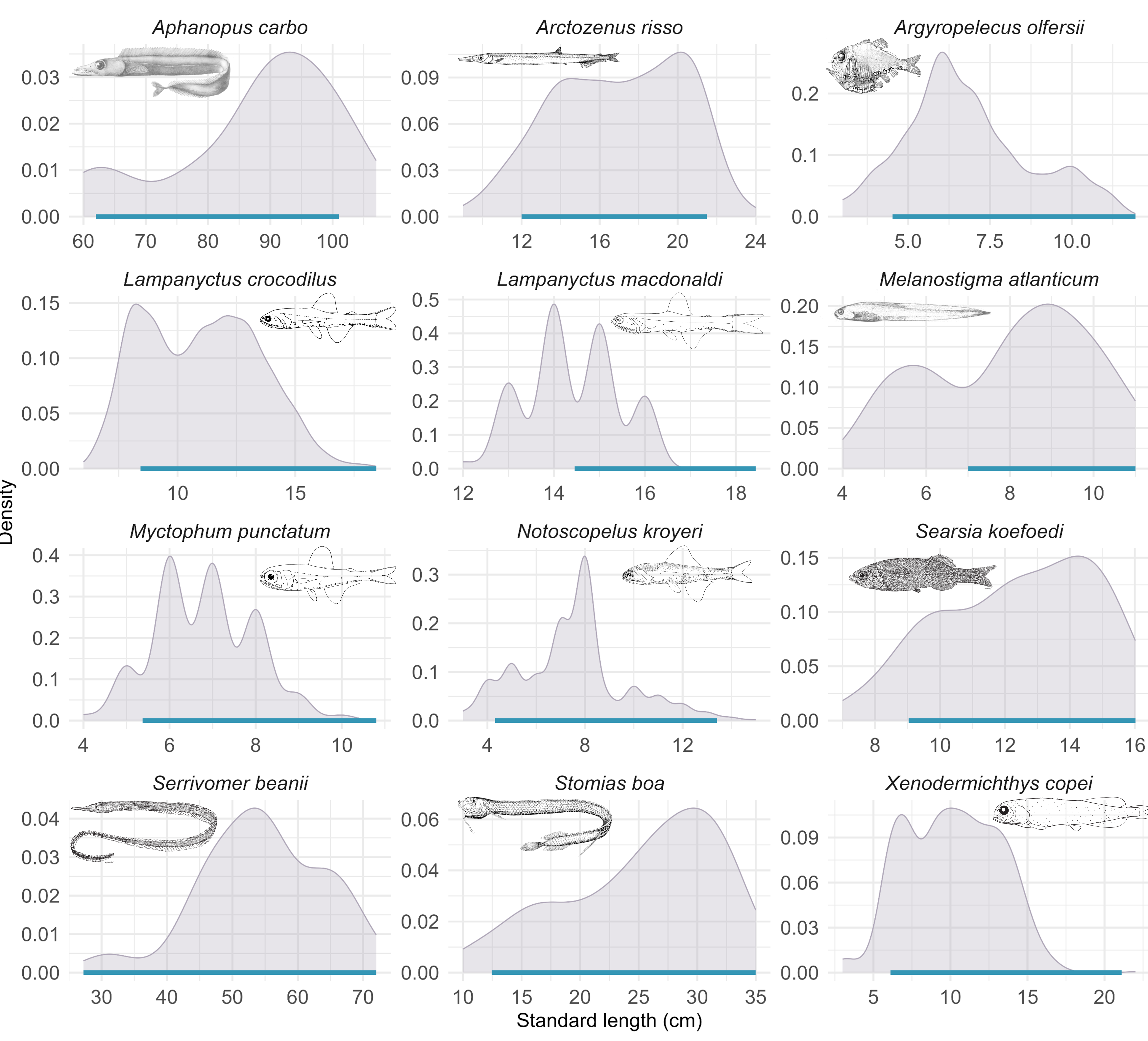

Supplement: Supplementary file 1 — Figure S1 [file ECE3-14-e11129-s003.tif]

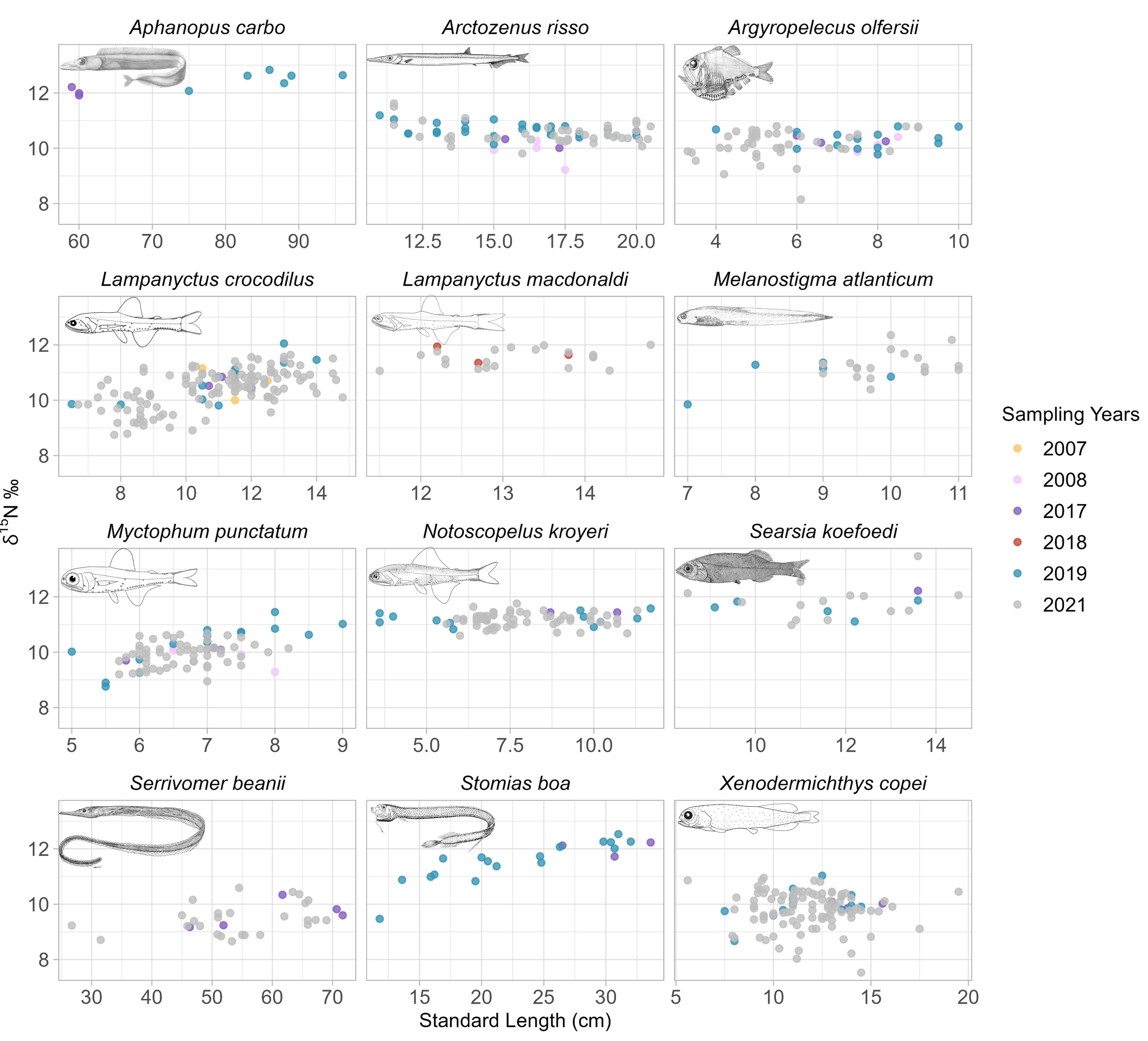

Supplement: Supplementary file 2 — Figure S2 [file ECE3-14-e11129-s002.tif]
